# *DNA*: detecting early signs of neurodegenerative diseases through activity and sleep analysis

**DOI:** 10.3389/fnins.2025.1617758

**Published:** 2025-08-19

**Authors:** Hasina Attaullah, Sanaullah Sanaullah, Annika Peters, Qazi Arbab Ahmed, Justin Baudisch, Thorsten Jungeblut

**Affiliations:** Resource-Efficient Microelectronics and Cognitive Edge Computing Research Group, Bielefeld University of Applied Sciences and Arts, Bielefeld, Germany

**Keywords:** smart homes, neurodegenerative disease, sleep pattern, cognitive decline, motor decline, AI, neurosciences

## Abstract

Early detection of neurodegenerative diseases, such as Alzheimers and Parkinsons, is essential for timely intervention, which can improve patients' quality of life and slow down disease progression. Traditional diagnostic methods rely heavily on clinical tests, which can be infrequent and may not capture slight behavioral changes that indicate early cognitive or motor decline. This work presents a novel approach using smart home data to detect early signs of neurodegeneration through continuous monitoring of sleep patterns and daily activity routines. In a smart home environment, sensors passively monitor daily routines, sleep quality, and mobility patterns of the elderly persons. This paper introduces a novel framework combining the Sleep Deviation Patterns (SDP) and the Weighted Activity Deviation Index (WADI) to comprehensively analyze deviations in sleep and daily routines. The SDP framework captures deviations in sleep onset, duration, interruptions, and consistency using metrics such as Sleep Onset Deviation, Sleep Duration Deviation, Sleep Interruption Index, and Sleep Consistency Index–aggregated into a weighted Sleep Deviation Score. WADI quantifies deviations in daily activities by computing weighted absolute deviations of activity proportions relative to a reference routine. Thus, we applied our framework to real-world smart home datasets (TM001-TM004) from the CASAS project, which include labeled activity data from both single- and multi-resident households. Experimental findings reveal a distinct stratification: TM001 and TM002 exhibited Low WADI and Low SDP with an average of >0.015 − >0.2 scores, suggesting stable routines, whereas TM003 and TM004 demonstrated elevated with an average of >0.03 − >0.4 values, indicating disrupted behaviors. In TM004, up to 28% of days were flagged as anomalous, correlating with patterns consistent with early neurodegeneration such as fragmented sleep and disorganized activity routines. Finally, experimental results demonstrate that the combined SDP and WADI frameworks effectively identify irregularities in sleep and activity patterns on real-world datasets. The proposed approach offers a robust and scalable solution for health monitoring, with potential applications in neurodegenerative disease detection, personalized healthcare, and smart home systems.

## 1 Introduction

Neurodegenerative diseases, including Alzheimers, Parkinsons, and other forms of dementia, are a growing public health challenge, particularly in aging populations (Batista and Pereira, 2016). These conditions lead to a gradual decline in cognitive, motor, and emotional functions, significantly impacting the quality of life for both patients and caregivers. Early detection of neurodegenerative diseases can be crucial as it allows for timely interventions, potentially slowing disease progression, allowing patients to make lifestyle and care decisions in the early stages of decline. Traditional diagnostic approaches, such as cognitive tests, neuroimaging, and patient-reported symptoms, often detect the disease only after substantial neurological damage has occurred, limiting the effectiveness of therapeutic interventions (Tur et al., 2018). Thus, there is a pressing need for innovative, non-invasive methods to detect the early signs of neurodegenerative diseases.

Sleep disturbances and changes in daily activities are some of the earliest symptoms in several neurodegenerative conditions. In patients with Parkinson's disease, for instance, rapid eye movement (REM) sleep behavior disorder often appears years before motor symptoms, serving as an early indicator (Tekriwal et al., 2017). Similarly, disruptions in sleep efficiency, increased wake after sleep onset, and fragmented sleep cycles are commonly observed in Alzheimers disease (Peter-Derex et al., 2015) and other forms of dementia (Shi et al., 2018). Beyond sleep, changes in daily routines such as reduced mobility, irregular activity patterns, and extended periods of sedentary behavior can suggest cognitive and motor impairments. Unobtrusive, non-wearable sensors, in particular, offer a promising approach to monitor these changes without relying on patients to wear devices, which can be a challenge, especially for those with dementia who may forget to wear them (Alharbi, 2022; Ó Breasail et al., 2021). While there are limitations to using such sensors for sleep rhythm testing, activities like nighttime wandering or stove usage can serve as strong indicators of sleep disturbances and other behavioral changes in dementia patients. Tracking these indicators in real-time can provide a continuous picture of a person's functional status, allowing healthcare providers to identify deviations from baseline behaviors that may signal early neurodegeneration. Therefore, this study aims to bridge the gap between traditional diagnostic approaches and AI-driven behavioral monitoring, using only smart home data to enhance early detection. The smart home data was collected unobtrusively by sensors installed in the home. This ensured that the daily routines of the person living there were not disturbed.

The main contributions of this paper are threefold, which are as follows:

- We present a comprehensive survey of neurodegenerative diseases, including their types, prevalence, facts and figures, underlying causes, and the latest insights into their progression and impact on global health.
- We develop a predictive model for the early detection of neurodegenerative symptoms based on sleep and activity patterns using smart home data.
- We evaluate the efficacy of smart home-based monitoring in identifying subtle behavioral changes indicative of neurodegeneration.

The rest of the paper is organized as follows: Section 1 concludes by highlighting the importance of understanding sleep and daily activity patterns for early detection of neurodegenerative diseases. These diseases, including Alzheimer's, Parkinson's, and other forms of cognitive and motor decline, often manifest subtle changes in behavior and routines that can be quantitatively analyzed.

In Section 2, we provide a detailed discussion of neurodegenerative diseases, covering their causes, diagnostic approaches, progression stages, and currently available treatments, supplemented by relevant facts and figures to provide a comprehensive background for this study, followed by the literature review in Section 3. Section 4 outlines the proposed approach, detailing the methodology behind the WADI and SDP frameworks. Section 5 presents the experimental findings, evaluating the effectiveness of these methods in detecting early behavioral deviations associated with neurodegenerative diseases. Finally, Section 6 summarizes the key insights, discusses the implications of the study, and highlights potential directions for future research.

## 2 Background

Neurodegenerative diseases, such as Alzheimer's and Parkinson's, are progressive disorders affecting millions of people worldwide, with cases expected to triple by 2050. These conditions often begin with subtle changes in sleep patterns and daily activities, making early detection crucial. Advances in AI, data analytics, and smart home technologies have enabled non-invasive monitoring of these behavioral changes, offering a pathway to timely intervention and improved outcomes. This section outlines the causes, diagnostic methods, progression stages, and treatments for these diseases, providing the foundation for the proposed methodologies.

### 2.1 Causes of neurodegenerative diseases

Neurodegenerative diseases can be caused by a variety of factors, including:

- Sleep Deviation Patterns (SDP) (Shen et al., 2023): The SDP framework–comprising onset deviation, duration, interruptions, and consistency–captures sleep irregularities that are clinically linked to early neurodegeneration. Additionally, by Anghel et al. (2023), disrupted sleep onset and fragmentation are associated with circadian dysfunction in Alzheimers, while REM disturbances and frequent awakenings are early signs of Parkinsons and dementia with Lewy bodies. These patterns reflect changes in brain regions regulating sleep-wake cycles, supporting SDP's relevance for early detection.
- Genetics (Baig et al., 2020): Genetics models play a significant role in the development of neurodegenerative diseases. Conditions like Huntington's disease and Alzheimer's are directly linked to inherited genetic mutations, which increase the likelihood of disease onset. In these cases, specific gene alterations trigger the progressive degeneration of neurons, leading to cognitive and motor impairments over time Pihlstrøm et al. (2018).
- Age (Hou et al., 2019): Age is the strongest known risk factor for neurodegenerative diseases. The risk increases significantly as people age, particularly after the age of 65.
- Environmental factors (Chin-Chan et al., 2015): Exposure to pesticides, air pollution, and heavy metals has been linked to neurodegenerative diseases.
- Lifestyle Factors:

- Diet and Nutrition: Diets high in saturated fats and low in antioxidants may increase the risk of diseases like Alzheimer's and Parkinson's. On the other hand, following a Mediterranean diet that emphasizes fruits, vegetables, whole grains, healthy fats, and lean proteins has been associated with a lower risk of these conditions, likely due to its anti-inflammatory and neuroprotective properties.
- Physical Inactivity: A sedentary lifestyle and lack of regular physical activity have been linked to a higher risk of developing neurodegenerative diseases. Exercise plays an important role in maintaining brain health by promoting blood flow, reducing inflammation, and supporting neural plasticity. Studies suggest that individuals who engage in regular physical activity have a lower likelihood of cognitive decline, as exercise helps protect neurons and enhances overall brain function.

- Vascular Health: Conditions such as hypertension, diabetes, and high cholesterol can contribute to vascular damage in the brain, which is linked to an increased risk of diseases like vascular dementia and Alzheimer's disease (O. Akinyemi et al., 2013).
- Other diseases: Conditions like cardiovascular diseases can impact blood flow to the brain, contributing to the risk of neurodegeneration (Dickstein et al., 2010).

### 2.2 Common neurodegenerative diseases

- Alzheimers Disease (AD): Characterized by the deterioration of cognitive function and synaptic pathology. Sleep disturbances are highly prevalent in individuals with Alzheimers, manifesting as reduced sleep efficiency, frequent nighttime awakenings, and fragmented sleep. Studies suggest that poor sleep contributes to the accumulation of amyloid-beta, a protein linked to Alzheimer's, creating a cyclical relationship between sleep disruptions and neurodegeneration. Blackwell and Yaffe (2014) highlighted that individuals with fragmented sleep patterns have an increased risk of developing Alzheimers, suggesting that sleep quality could serve as an early indicator of cognitive decline.
- Parkinsons Disease (PD): Identified by the loss of dopamine-producing neurons in the brain and characterized by motor symptoms like tremors, stiffness, and balance problems. Parkinsons patients frequently experience REM Sleep Behavior Disorder (RBD), a condition characterized by the acting out of dreams during REM sleep. Research shows that RBD often appears years before motor symptoms, making it one of the earliest observable markers of PD. Postuma et al. (2012) demonstrated that nearly 80% of individuals with RBD eventually develop a neurodegenerative disease, with PD being the most common. Such findings highlight the diagnostic value of sleep patterns in predicting neurodegenerative diseases.
- Multiple Sclerosis: Multiple Sclerosis (MS) is a chronic autoimmune disorder in which the immune system mistakenly attacks the myelin sheath, the protective covering of nerve fibers. This disruption affects nerve signal transmission, leading to early symptoms such as fatigue, difficulty walking, and cognitive impairments (Compston and Coles, 2008). Research suggests that changes in sleep patterns and daily activity levels, such as frequent nighttime awakenings and reduced physical movement, may indicate disease progression. Continuous monitoring of these behavioral changes through smart home sensors or wearable devices could assist in early detection and timely medical intervention.
- Huntingtons Disease: Huntingtons Disease (HD) is a genetic neurodegenerative disorder caused by a mutation in the huntingtin gene, which leads to progressive cognitive decline, psychiatric symptoms, and involuntary movements (Ross and Tabrizi, 2011). One of the earliest indicators of the disease is disrupted sleep patterns, including reduced REM sleep and frequent nighttime awakenings. Additionally, individuals may experience changes in daily routines, such as irregular physical activity levels or extended periods of inactivity. By continuously monitoring these subtle behavioral shifts, it is possible to detect early signs of Huntingtons Disease.

### 2.3 Diagnosis of neurodegenerative diseases

The diagnosis of neurodegenerative diseases has significantly evolved, incorporating both traditional medical evaluations and modern technological advancements to improve accuracy and early detection. This multidisciplinary approach integrates clinical evaluation, neuroimaging, biomarker analysis, genetic testing, and unfolding advances in digital health.

- Clinical Evaluation (Erkkinen et al., 2018): Neurodegenerative disorders are primarily diagnosed based on clinical assessment. For the assessment of cognitive function, motor function, reflexes, and sensory perception, neurologists initially assess the patient's symptoms and medical history prior to conducting a comprehensive physical examination. Cognitive impairment in its early stages is typically identified by the application of cognitive screening tools such as the Montreal Cognitive Assessment (MoCA) and the Mini-Mental State Examination (MMSE).
- Neuroimaging: Neuroimaging is a very important method for reading structural and functional changes that are linked with neurodegenerative disease (Jack et al., 2018). Two medical imaging exams, CT scans and MRIs, help doctors detect problems that may be related to how a disease is progressing, damage to blood vessels, or loss of brain tissue can cause a brain tumor (Sanaullah and Jungeblut, 2023). Positron Emission Tomography (PET) scans can identify biochemical changes, including dopaminergic deficits in Parkinson's disease and the presence of amyloid-beta plaques in Alzheimer's disease. This capability enables monitoring of disease progression and also accelerates diagnosis.
- Biomarkers: Biomarker analysis is essential for the early identification and detection of neurodegenerative disease, especially in blood and cerebrospinal fluid (CSF) (Thijssen et al., 2020). Alzheimer's disease is characterized by biomarkers including decreased amyloid-beta 42 and increased tau protein levels in CSF. Alpha-synuclein aggregation is observable in Parkinson's disease.
- Genetic Testing: Genetic testing (Bertram and Tanzi, 2005; Xue, 2019) is increasingly used in the diagnosis of hereditary neurodegenerative conditions like Huntingtons disease, familial Alzheimers disease, and familial amyotrophic lateral sclerosis (ALS). Techniques such as next-generation sequencing (NGS) enable the identification of specific mutations, including expansions in the HTT gene for Huntingtons or mutations in APP, PSEN1, and PSEN2 for Alzheimers.
- Emerging Technologies: The integration of digital health tools has revolutionized the diagnosis and monitoring of neurodegenerative diseases. Wearable devices, such as smartwatches and activity trackers, monitor motor symptoms, sleep patterns, and daily routines, offering real-time data to detect early changes. Speech analysis software, using smartphones or home devices, captures subtle variations in speech patterns linked to cognitive decline. Additionally, ambient sensor systems in smart homes track activity levels and anomalies, helping to identify signs of diseases like Alzheimers or Parkinsons.

### 2.4 Stages of neurodegenerative diseases

The progression of neurodegenerative diseases is generally divided into stages:

- Early Stage: Symptoms are mild and may include forgetfulness, slight muscle stiffness, or tremors.
- Middle Stage: Symptoms become more pronounced and interfere with daily activities. In diseases like Alzheimers, patients may experience significant memory lapses.
- Late Stage: Individuals may lose the ability to communicate effectively and require full-time care. Mobility may also be severely restricted.

### 2.5 Treatment and management

While there is no cure for most neurodegenerative diseases, treatment focuses on managing symptoms and maintaining quality of life:

- Medications: Drugs like cholinesterase inhibitors for AD and levodopa for PD are used to manage symptoms.
- Physical therapy: It helps maintain mobility and balance.
- Occupational therapy: It assists individuals in performing daily activities.
- Cognitive Therapies: Such as memory aids can help manage cognitive deficits.

### 2.6 Facts and figures

- Worldwide: Over 50 million people globally suffer from dementia, with Alzheimer's disease making up a significant portion of these cases. This statistic is sourced from Alzheimer's Disease International (ADI) (Alzheimer's Disease International, 2022), which regularly reports on global dementia figures.
- United States: Approximately 6.2 million Americans aged 65 and older are currently living with Alzheimer's. This data comes from the 2021 Alzheimer's Disease Facts and Figures report by the Alzheimer's Association (Alzheimer's Association, 2021).
- Canada: Over 747,000 Canadians are living with Alzheimer's and other dementias, as reported by the Alzheimer Society of Canada [Alzheimer Society of Canada (Alzheimer Society of Canada, 2021)].
- United Kingdom: Around 850,000 people are affected by dementia, predominantly Alzheimers, according to the Alzheimers Society UK (Alzheimer's Disease International, 2022).
- Germany: According to the Deutsche Alzheimer Gesellschaft (Deutsche Alzheimer Gesellschaft, 2023), approximately 1.8 million people in Germany are living with dementia as of the end of 2023. In 2023, about 445,000 individuals aged 65 and older were newly diagnosed with dementia. Projections indicate that, without significant advancements in prevention or treatment, the number of affected individuals aged 65 and above could rise to up to 2.7 million by 2050.
- France: According to the France Alzheimer Annual Report 2023 (France Alzheimer, 2023), over 1.2 million individuals in France are affected by Alzheimer's disease or related dementias. This number is projected to rise, with approximately 225,000 new cases diagnosed each year.
- China: China has the highest number of Alzheimers patients of any nation, with about 10 million cases, as reported by the Alzheimer's Disease Chinese (ADC) (Alzheimer's Disease Chinese (ADC), 2020).
- Japan: Around 5 million people are living with Alzheimers disease, reflecting its significant aging population, detailed by the Japanese Ministry of Health, Labour and Welfare (Japanese Ministry of Health, Labour and Welfare).
- India: A 2023 study published in Alzheimer's & Dementia: The Journal of the Alzheimer's Association (Lee et al., 2023) estimates that approximately 8.8 million Indians aged 60 and above are living with dementia, reflecting a prevalence rate of 7.4% in this age group.
- Brazil: There are around 1.2 million cases, with growing numbers due to an increasing elderly population, as reported by the Brazilian Alzheimer's Association (Associao Brasileira de Alzheimer (ABRAz), 2023).
- Australia: As of 2024, it is estimated that over 421,000 Australians are living with dementia. Without a medical breakthrough, this number is projected to rise to more than 812,500 by 2054, representing a 93% increase, based on reports from Dementia Australia (Dementia Australia, 2023).

The global prevalence of Alzheimers disease is expected to rise, especially in low and middle-income countries. The Alzheimer's Association projects (Alzheimer's Association, 2021) that by 2050, the number of people aged 65 and older with Alzheimer's disease may nearly triple, from 6.2 million to a projected 14 million in the United States alone, highlighting an urgent need for global healthcare planning and research. To address the rising prevalence of Alzheimers disease, innovative solutions like smart home technologies offer promising opportunities for early detection and care. Smart home technologies present a promising solution by providing continuous, passive monitoring of daily behaviors in naturalistic settings without disrupting daily routines with in-person or online assessments. The advantage of non-wearable sensors is that a person does not have to remember to wear (e.g., bracelets or rings). These technologies utilize non-intrusive sensors embedded within the home environment, such as motion detectors, door sensors, sleep trackers, and appliance usage monitors, to capture data on individuals' activities, movement patterns, and sleep quality. Such sensors offer the opportunity to detect early behavioral changes, often subtle and gradual, that might be missed in sporadic clinical assessments. For example, disruptions in sleep patterns, prolonged inactivity, and variations in daily routines can signal early neurodegenerative symptoms, providing valuable information for preventive care. Utilizing smart home data could enhance our ability to recognize early-stage symptoms of neurodegeneration, offering a path toward scalable, at-home healthcare solutions for at-risk populations.

## 3 Conceptual framework

**Figure 1:**
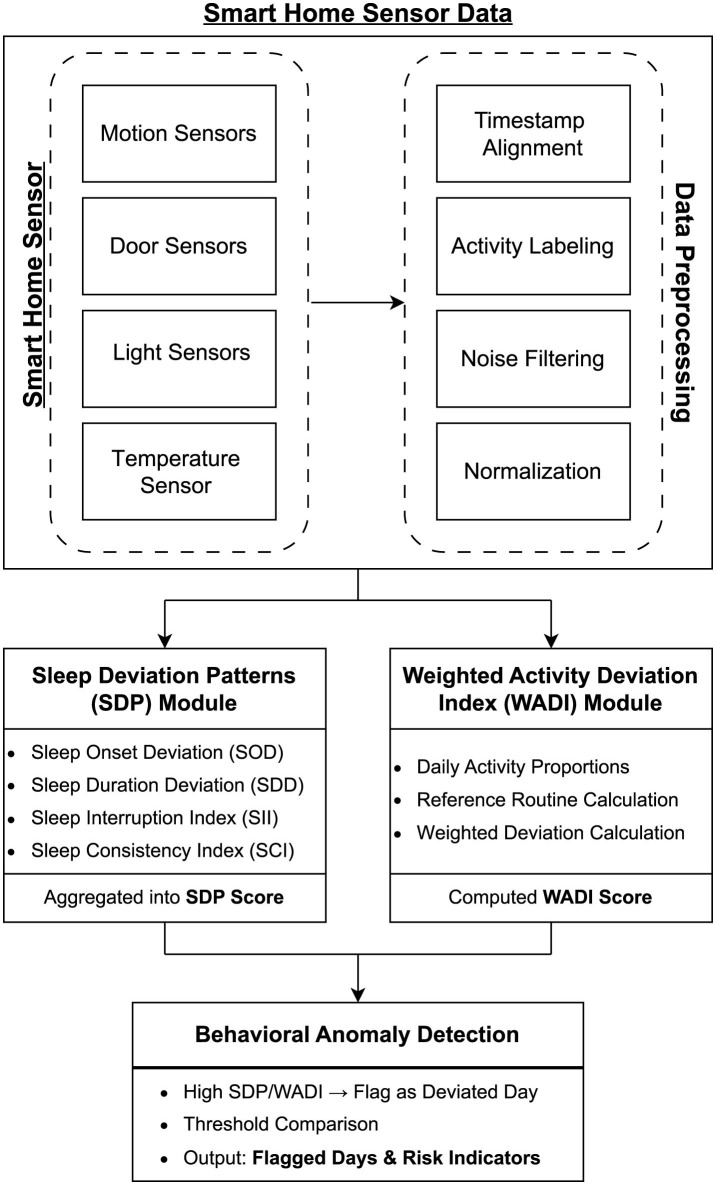
Block diagram of the proposed DNA framework showing the processing pipeline from smart home sensor data to behavioral anomaly detection using the SDP and WADI modules.

The early detection of neurodegenerative diseases has gained significant research attention due to their potential to delay symptom progression, improve quality of life, and reduce healthcare burdens. Traditional diagnostic methods, such as cognitive assessments and neuroimaging, are costly, require specialized equipment, and often detect neurodegenerative conditions only after significant cognitive or motor decline has occurred. To address these limitations, researchers are exploring passive, non-invasive monitoring solutions that leverage smart home technologies to capture behavioral markers indicative of early disease onset. This section reviews relevant literature on sleep disturbances and changes in daily activities as early indicators of neurodegenerative diseases, as well as machine learning techniques applied to smart home data for early detection. Furthermore, the overall flow of the proposed DNA framework—from sensor data collection to behavioral anomaly detection—is illustrated in Figure 1.

### 3.1 Wearable and sensor-based approaches for early detection

Recent studies have demonstrated the feasibility of wearable and ambient sensors for monitoring neurodegeneration. A study in the Journal of Parkinsons Disease (Smith et al., 2022) examined wrist-worn sensors for detecting subtle motor impairments in individuals at risk of Parkinsons disease. The sensors continuously monitored movement patterns and applied algorithms to detect early-stage motor dysfunction, which may precede clinical symptoms. Similarly, research published in Alzheimers & Dementia: The Journal of the Alzheimers Association (Min et al., 2025) focused on smartphone-based applications that assess cognitive function. These applications engage users in tasks designed to test memory, problem-solving, and attention–key areas affected in early-stage Alzheimers disease. By tracking performance over time, these applications help detect cognitive decline. Ambient sensors have also been used to measure daily routines and sleep patterns. One study published in the International Journal of Geriatric Psychiatry (Doe et al., 2021) explored using motion and sleep sensors to monitor older adults for the early detection of dementia. Findings indicated that interruption of sleep patterns and decreased physical movement levels were associated with cognitive impairment, demonstrating the potential of sensor technology for early disease detection. Yet, issues like privacy, behavioral differences, and model generalizability were raised, calling for more accuracy and ethical consideration.

### 3.2 Sleep monitoring as an indicator of cognitive decline

Sleep fragmentation and inconsistent sleep efficiency have been strongly associated with poorer cognitive performance in older adults. For instance, Lim et al. (2013) demonstrated that sleep fragmentation, measured accurately via actigraphy, was significantly associated with an increased risk of Alzheimers disease and faster cognitive decline. Kaneshwaran et al. (2019) further reported that sleep fragmentation correlates with cognitive impairment and microglial aging in both Alzheimers and non-Alzheimers groups. Similarly Sakal et al. (2023), found that variability in sleep efficiency predicts lower scores on cognitive tests such as the Digit Symbol Substitution Test (DSST) and verbal fluency in 65+ adults. Their findings coincided with newer results by Fjell and Walhovd (2024), the result showed disrupted sleep patterns, including fragmentation and shifts in sleep-wake states, predicted early signs of neurodegeneration and cognitive decline across different population groups. Blackwell and Yaffe (2014) showed how smart watches can observe sleep patterns and predict sleep disorders like insomnia and sleep REM disorders, all of which contribute to cognitive neurological conditions. They studied wearables for sleep monitoring and while they found it useful, but also pointed out problems about accuracy of the sensor measurements, compliance of the user, and environmental factors. Research on speech patterns is also being studied as a potential marker of cognitive decline. A study appeared in Computers in Biology and Medicine by Khodabakhsh et al. (2021) analyzed changes to speech, by using voice analysis software to observe speech changes when making phone calls. The findings linked variations in speech patterns to early-stage cognitive decline in Alzheimers patients, reinforcing the idea that behavioral biomarkers–whether in sleep, speech, or movement–can provide critical insights into neurodegeneration. A study in the Journal of Ambient Intelligence and Smart Environments Haghi et al., (2022) focused on using smart home data to predict critical health events such as falls and acute medical conditions. By analyzing motion sensor data, door contacts, and physiological parameters, the system was able to predict emergency events in elderly individuals, enabling early interventions.

### 3.3 Machine learning and clustering for behavioral analysis

Machine learning techniques have been widely used to analyze changes in sleep and activity patterns related to neurodegenerative diseases. (Shahid et al. 2022) employed K-means clustering on motion sensor data collected from elderly individuals over three years. The study found that shifts in sleep duration, nighttime awakenings, and wake-up times correlated with the onset of dementia and Parkinsons disease. The Smart Aging Monitoring and Early Dementia Recognition (SAMEDR) framework (Ghayvat and Gope, 2023) further explored the application of AI in categorizing cognitive impairment through sensor data analysis, focusing on activity recognition and behavioral monitoring. The role of seasonal variations in sleep patterns has also been studied. Research by (Shahid et al. 2024) emphasized the importance of distinguishing normal seasonal variations in sleep from disturbances caused by health conditions, which could provide additional insights into early diagnosis. However, challenges such as flaws in sensory datasets, data reliability, and the availability of labeled datasets for machine learning models were identified. The Smarter Safer Homes (SSH) platform (Prabhu et al., 2023) demonstrated that motion sensor data can track behavioral changes in older adults, particularly those experiencing depression, a condition that often coexists with neurodegenerative disorders. Similarly, research on forecasting activities of daily living (ADL) in elderly smart homes (Shahid et al., 2023) utilized Long Short-Term Memory (LSTM) models for predicting ADLs and Mahalanobis distance for anomaly detection. The study identified abnormal behaviors related to prolonged inactivity and unusual movement patterns, which may serve as early indicators of cognitive and physical health deterioration.

### 3.4 Daily activity monitoring and routine consistency in neurodegeneration

Activity patterns and mobility are closely linked to cognitive health. Changes in daily routines, movement frequency, and transitions between home spaces can provide valuable indicators of neurodegenerative diseases. (Reed et al. 2016) examined the impact of declining Activities of Daily Living (ADLs) on caregiver burden and healthcare costs, emphasizing the need for early interventions to reduce long-term challenges. The study found that impairments in mobility, hygiene, and household management correlated with increased caregiver time and financial strain. (Kaye et al. 2011) conducted research using motion sensors to track elderly individuals daily movement patterns. The study revealed that reductions in movement frequency, fewer room-to-room transitions, and increased sedentary behavior were predictive of early-stage neurodegenerative symptoms. These findings support the idea that continuous monitoring of mobility patterns through smart home sensors can provide early warnings for cognitive decline. A study by (Wu et al. 2021) focused on the use of smart home sensor data to monitor ADLs in elderly individuals, particularly those with dementia. The research explored the integration of multi-sensor data streams to detect health changes and enable early interventions. Although the study primarily focused on general activity monitoring, its findings suggest that smart home technologies could play a role in detecting behavioral changes associated with neurodegenerative diseases.

The existing body of research highlights the potential of smart home sensor technologies as a non-intrusive and continuous monitoring system capable of detecting early signs of neurodegenerative diseases. By analyzing patterns in sleep and daily activities, these systems can provide valuable insights into behavioral changes that may indicate cognitive decline, allowing for timely medical interventions and improved quality of life for the elderly.

Despite these advancements, most current approaches focus solely on either activity recognition or sleep monitoring rather than integrating both for a comprehensive assessment of behavioral patterns. Generalizability remains a concern, as models trained on specific populations may not be effective across individuals with diverse routines and lifestyles. Furthermore, most existing methods employ adaptive or personalized thresholding techniques, which, while beneficial, introduce variability and require extensive calibration for each individual.

To address these limitations, we propose DNA (Detecting Early Signs of Neurodegenerative Diseases through Activity and Sleep Analysis), a framework that integrates both activity-based and sleep-based anomaly detection. Using the WADI and SDP, this approach offers a structured, data-driven method to quantify deviations in daily routines and sleep behaviors. The next section outlines the methodology behind the proposed framework, explaining how it is designed to identify behavioral irregularities that may serve as early indicators of neurodegenerative diseases.

## 4 Methodology

The increasing merger of artificial intelligence, data analytics, and smart home technologies has created new possibilities for the non-invasive monitoring of sleep patterns and daily activities. Such technologies enable researchers to track changes in behavior and detect deviations from an individual's usual routine, which are early signs of neurodegenerative disorders. By detecting such patterns early, one can initiate interventions earlier, thereby delaying the progression of diseases and enhancing the quality of patient care overall.

To bridge this gap, we propose DNA (Detecting Early Signs of Neurodegenerative Diseases through Activity and Sleep Analysis), a new framework that evaluates daily activity and sleep systematically. The following sections detail the design and implementation of this proposed framework, illustrating how it works to provide a robust, non-invasive method for detecting early signs of neurodegeneration through smart home sensor data.

### 4.1 Weighted Activity Deviation Index

The WADI is designed to track changes in an individuals daily activity patterns and compare them against their usual routine. Unlike conventional methods that treat all activities equally, WADI assigns weights to different activities based on their significance in a persons daily schedule. This ensures that deviations in key activities—such as sleep, eating, or mobility—are given greater importance when assessing behavioral changes. By capturing even subtle shifts in routine, WADI enables early intervention and provides a more reliable measure of long-term behavioral trends that may indicate cognitive decline or neurodegenerative progression.

#### 4.1.1 Definition

WADI quantifies the weighted sum of absolute deviations between the normalized daily activity proportions and the normalized reference activity proportions.


(1) $$\begin{array}{l} \text{WADI}=\overset{n}{\underset{i=1}{\sum }}w_{i}\cdot |A_{i}-R_{i}| \end{array}$$

Where: - *w*_*i*_: Weight assigned to the *i*^*th*^ activity based on its importance. All weights satisfy:


(2) $$\begin{array}{l} \overset{n}{\underset{i=1}{\sum }}w_{i}=1 \end{array}$$

*A*_*i*_: Proportion of time spent on the *i*^*th*^ activity in the daily routine being analyzed. It is computed as:


$$A_{i}=\frac{\text{Time spent on activity}i}{\text{Total time spent on all activities on that day}}$$

- *R*_*i*_: Proportion of time spent on the *i*^*th*^ activity in the reference routine, calculated as:


$$R_{i}=\frac{\text{Average time spent on activity}i\text{across all days}}{\text{Total time spent on all activities in the reference}}$$

- *n*: Total number of activities.

#### 4.1.2 Key properties

- Activities that have more critical (e.g., sleep, personal hygiene) are assigned higher weights (*w*_*i*_). Less significant activities (e.g., entertaining guests) receive lower weights.
- Absolute deviation: the use of absolute differences ensures that both underperformance and overperformance in activities are penalized equally.
- Reference routine: the reference routine can be tailored to the application, such as the mean routine, a predefined healthy routine, or an individual's baseline routine.

#### 4.1.3 Application workflow

- Activity data collection: log time spent on various activities daily using sensors or manual reports.
- Normalization: convert activity durations into proportions for both the daily routine and the reference routine.
- Weight assignment: assign weights (*w*_*i*_) to activities based on their relevance to the context.
- Deviation calculation: compute deviations (|*A*_*i*_−*R*_*i*_|) for each activity.
- WADI computation: aggregate weighted deviations across all activities to compute WADI.

#### 4.1.4 Interpretation

- Lower WADI values: indicate closer alignment between the daily routine and the reference routine. Suggest stable, predictable behavior.
- Higher WADI values: indicate significant deviations from the reference routine. Suggest potential behavioral anomalies or disruptions.

#### 4.1.5 Example implementation

**Example weights:** WADI computation for a sample day:

**Reference routine:**


(3) $$\begin{array}{l} \;R_{\text{Sleep}}=0.30,\;R_{\text{Personal\_Hygiene}}=0.10,\\ R_{\text{Cook\_Breakfast}}=0.05 \end{array}$$

**Daily routine:**


(4) $$\begin{array}{l} \;A_{\text{Sleep}}=0.25,\;A_{\text{Personal\_Hygiene}}=0.12,\\ A_{\text{Cook\_Breakfast}}=0.10 \end{array}$$

**WADI calculation:**


(5) $$\begin{array}{l} \text{WADI}=\left(0.25\cdot |0.25-0.30|\right)\\ \;+\left(0.10\cdot |0.12-0.10|\right)\\ \;+\left(0.08\cdot |0.10-0.05|\right) \end{array}$$

**Simplified WADI:**


(6) $$\begin{array}{l} \text{WADI}=\left(0.25\cdot 0.05\right)+\left(0.10\cdot 0.02\right)\\ \;+\left(0.08\cdot 0.05\right) \end{array}$$

**Final WADI:**


(7) $$\begin{array}{l} \text{WADI}=0.0125+0.002+0.004\\ \;=0.0185 \end{array}$$

### 4.2 Sleep deviation patterns

SDP is a structured approach for monitoring sleep patterns, capturing irregularities such as variations in sleep onset, duration, interruptions, and consistency. By integrating these two models, DNA provides a comprehensive assessment of behavioral anomalies, allowing for earlier detection of neurodegenerative decline. The following sections provide a detailed explanation of the methodology behind SDP, including its formulation, implementation, and potential applications. The SDP framework evaluates deviations in sleep behavior from a reference routine. It goes beyond simple duration tracking by incorporating sleep onset, interruptions, consistency, and quality metrics to quantify deviations. The **SDP framework** focuses on analyzing deviations in sleep behaviors using four key metrics:

1. **Sleep onset deviation (SOD)**:
  $$\text{SOD}=\min\left(\left|O-R_{O}|,24-|O-R_{O}\right|\right)$$
  where *O* is the observed onset time and *R*_*O*_ is the reference onset time.
2. **Sleep duration deviation (SDD)**:
  $$\text{SDD}=\frac{\left|D-R_{D}\right|}{3600}$$
  where *D* is the observed sleep duration (in seconds) and *R*_*D*_ is the reference duration.
3. **Sleep Interruption Index (SII)**:
  $$\text{SII}=\frac{I}{D}$$
  where *I* is the number of interruptions and *D* is the sleep duration (in hours).
4. **Sleep Consistency Index (SCI)**:
  $$\text{SCI}=\max\left(0,1-\frac{\sigma_{O}}{24}-\frac{\sigma_{D}}{R_{D}}\right)$$
  where σ_*O*_ and σ_*D*_ are the standard deviations of sleep onset and duration, respectively.

These metrics are aggregated into a single **SDP score** using weighted contributions:


$$\text{SDP}=w_{1}\cdot \text{SOD}+w_{2}\cdot \text{SDD}+w_{3}\cdot \text{SII}+w_{4}\cdot \left(1-\text{SCI}\right)$$

where *w*_1_, *w*_2_, *w*_3_, and *w*_4_ are the weights assigned to each metric.

The WADI and SDP frameworks are applied independently to assess daily routines and sleep patterns. Anomalous days, flagged by high WADI or SDP scores, are analyzed to identify potential behavioral changes indicative of cognitive or motor decline. These frameworks enable a holistic assessment of deviations, integrating daily activity and sleep behaviors into a unified analysis. The interpretation of SDP score is as follows:

- Lower SDP score indicates better alignment with the reference routine.
- Higher SDP scor reflects significant deviations, suggesting potential issues such as irregular sleep or interruptions.

### 4.3 Algorithms

In Algorithm 1, the WADI quantifies significant changes in daily activity patterns by relating a person's observed activities to an expected routine. Each activity has a weighted deviation that depicts the relative importance of that activity, each deviation is then computed based on that weight. To detect abnormal changes, the algorithm employs a thresholding mechanism to check for a day where the distribution of activity was significantly different than the expected routine. The algorithm uses a scaling factor, α to vary the sensitivity of the dynamic threshold. The greater the value of α the more conservative the flagging of deviations, while lower values make deviance detection more sensitive to change. Similarly, the SDP Algorithm 2 evaluates sleep irregularities by analyzing key sleep parameters, including variations in sleep onset, total sleep duration, interruptions, and consistency over time. By applying a weighted formula, the algorithm generates a cumulative sleep deviation score, helping to detect disruptions in circadian rhythms that may indicate early signs of neurological or physiological conditions.

**Algorithm 1:**
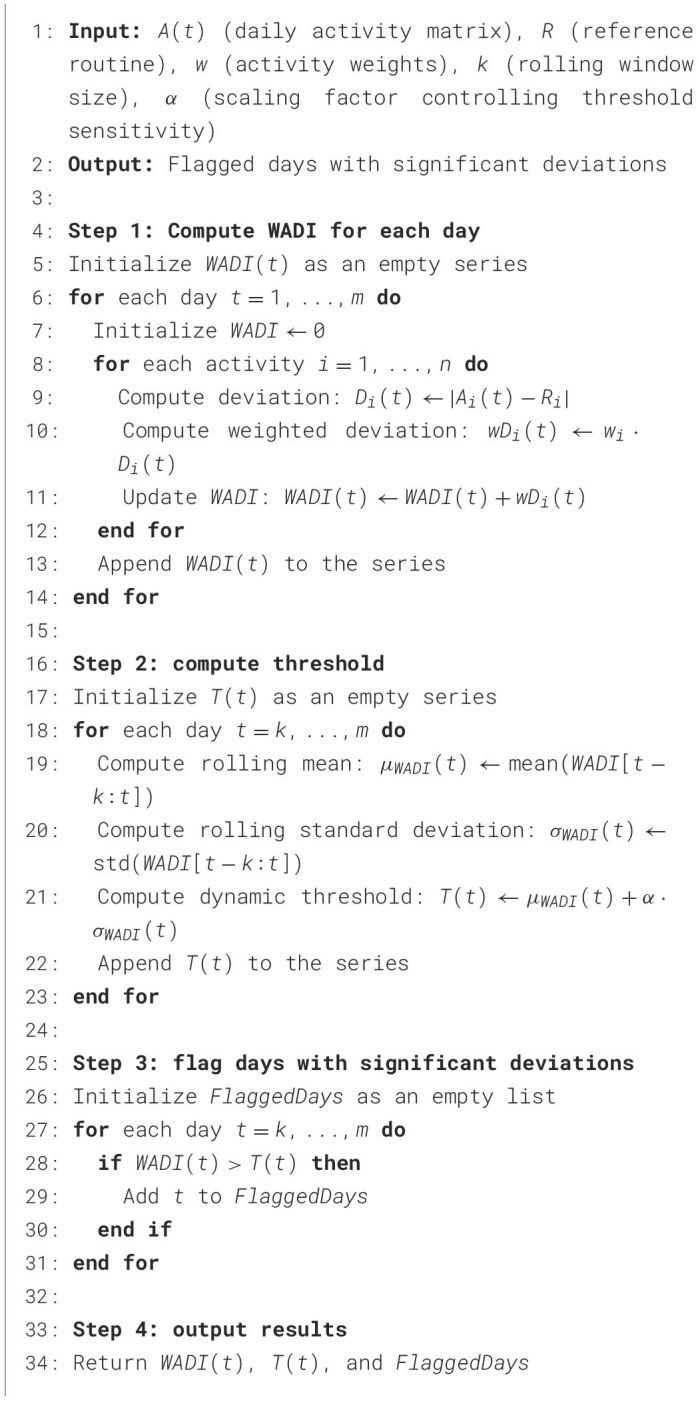
Dynamic Threshold-Based Weighted Activity Deviation Index (WADI)

**Algorithm 2:**
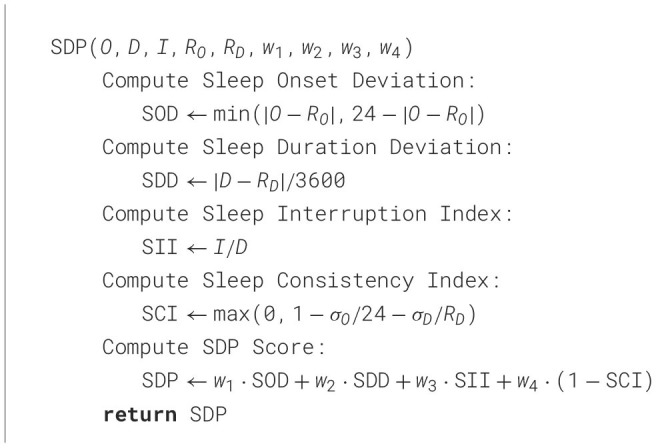
Sleep deviation patterns (SDP)

Both algorithms rely on statistical measures and threshold-based assessments to identify behavioral anomalies, making them effective tools for detecting potential health concerns through passive, non-invasive monitoring of daily activities and sleep behaviors.

## 5 Results and discussion

This research utilizes publicly available datasets collected on the CASAS (Cook et al., 2010) smart home project, namely the TM series datasets: TM001, TM002, TM003, and TM004. These datasets, which were gathered from older adults with chronic health issues, offer a deep understanding of the reflection of day-to-day interactions and behaviors in smart home settings. These are commonly utilized for activity recognition, anomaly detection, and behavior analysis studies, and thus are significant to the research of routine patterns and anomaly detection. The data is derived from real-world smart home sensor installations, offering a true reflection of real-world daily routines and activities in smart home environments.

### 5.1 Datasets overview

The TM series datasets capture the daily routines of individuals living in smart home environments, providing labeled activity data for research purposes.

- TM001, TM002, and TM003 focus on single-resident households, where activities such as sleeping, eating, and performing household chores are labeled to track behavioral patterns.
- TM004 differs from the previous datasets as it includes multiple residents, with sensor data labeled according to individual activities, making it a more complex dataset for analysis.

By incorporating both single- and multi-resident datasets, this study ensures a comprehensive understanding of daily activity patterns, which is essential for detecting behavioral deviations that may indicate early signs of neurodegenerative diseases.

### 5.2 Sensors used

The datasets use various sensors installed throughout the smart home to capture activity patterns. Key sensors include:

- Motion sensors: detect movement in specific areas (e.g., living room, bedroom, bathroom, and kitchen).
- Door sensors: capture the opening and closing of doors, such as bedroom doors or refrigerators.
- Temperature sensors: record ambient temperature changes in the home environment.
- Light sensors: detect changes in lighting.

### 5.3 Data structure

The data is recorded as timestamped events with the following attributes: Timestamp: The exact time of the event. Sensor ID: The unique identifier of the sensor triggering the event. Sensor Status: Indicates the sensor's state (e.g., ON/OFF for motion sensors, OPEN/CLOSE for door sensors). Activity Label: The annotated activity (e.g., Sleep, Cook, Eat), if available.

### 5.4 Processing and analysis

To derive meaningful insights, the raw sensor data was processed to extract:

- Daily activity durations: calculated by aggregating the total time spent on each labeled activity (e.g., sleeping, eating, relaxing).
- Sleep metrics: derived from activities like "Sleep" or "Bed_Toilet_Transition," capturing sleep onset, duration, and interruptions.
- Activity deviations: using the WADI framework, deviations in daily activity patterns were quantified concerning a reference routine.
- Sleep deviations: using the SDP framework, deviations in sleep behaviors, such as onset timing and interruptions, were calculated.

#### 5.4.1 Sleep deviations (SDP):

Sleep onset deviations were prominent on certain days, often correlating with late bedtimes or interruptions (e.g., “Bed_Toilet_Transition”). Increased interruptions during sleep were linked to higher SDP scores, highlighting potentially significant behavioral anomalies. The results demonstrate the utility of using sensor-based datasets to monitor deviations in daily routines and sleep patterns. However, to better understand the contextual factors behind these deviations (e.g., weekends or unusual events), continuous real-time data collection is essential.

### 5.5 Observations and initial results

In this section, we present the outcomes of our analyses based on the WADI, SDP, overlap detection, night bathroom activity counts, and long-duration activity monitoring. These results are from a thorough study of smart home sensor data that examined for unusual behaviors, sleep problems, and long periods of inactivity. The indices may point to neurodegenerative symptoms at a certain level. Each dataset is for a distinct person, and the variances in the WADI and SDP patterns probably show that their health, lifestyle, and potentially even their cognitive conditions are different.

#### 5.5.1 Low WADI & low SDP (stable activity & sleep)—Potentially healthy individuals

**Figure 2:**
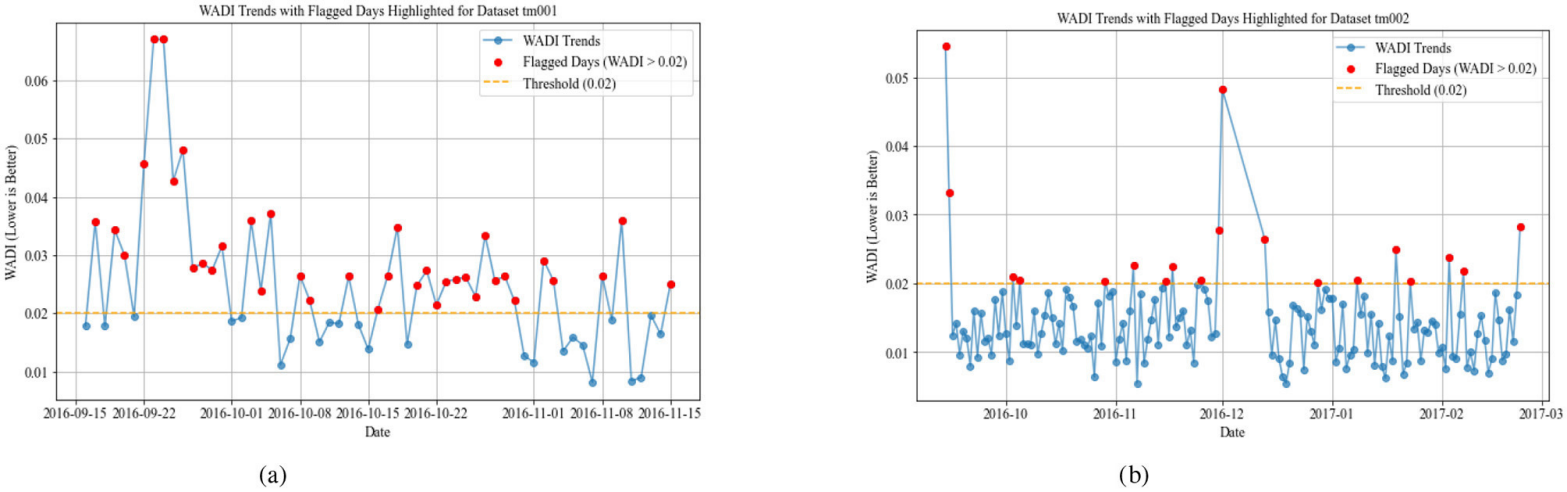
Weighted Average Deviations Index with a fixed threshold of 0.02 in datasets TM001 and TM002. **(a)** WADI for TM001. **(b)** WADI for TM002.

Individuals with low WADI values exhibit a structured and consistent daily routine with minimal deviations in their activity levels. This suggests a regular circadian rhythm, which is characteristic of cognitively healthy adults who maintain stable habits. Likewise, a low SDP score indicates consistent sleep onset, duration, and minimal nighttime disruptions, which is associated with good cognitive health. Figure 2 for dataset TM001 appears to have a stable daily routine and sleep schedule, suggesting a cognitively intact individual.

#### 5.5.2 Moderate WADI & SDP (mild irregularities)—Early cognitive impairment or age-related changes

The WADI result for dataset TM002 in Figure 2b exhibits moderate activity and sleep deviations, which could indicate mild cognitive impairment (MCI), early-stage neurodegenerative changes, or any other chronic illness. Older adults without dementia may still show some day-to-day variability in sleep and activity, but significant irregularities in both may suggest emerging symptoms.

#### 5.5.3 High WADI & high SDP (severe irregularities)—Possible signs of neurodegeneration

**Figure 3:**
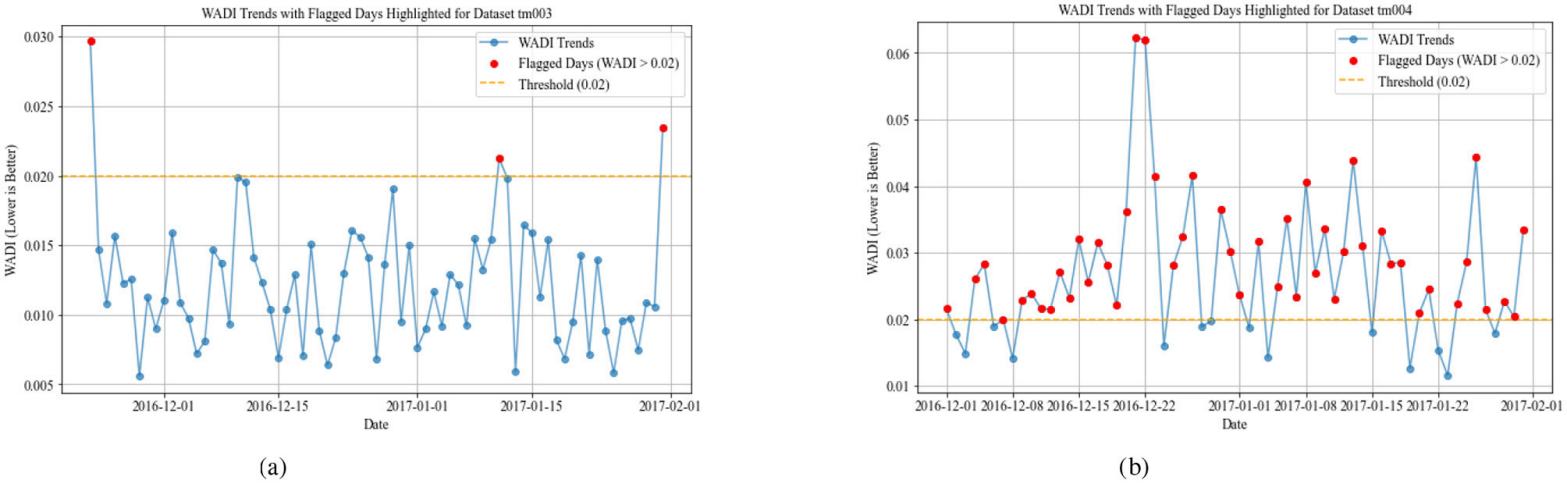
Weighted Average Deviations Index with a fixed threshold of 0.02 in datasets TM003 and TM004. **(a)** WADI for TM003. **(b)** WADI for TM004.

**Figure 4:**
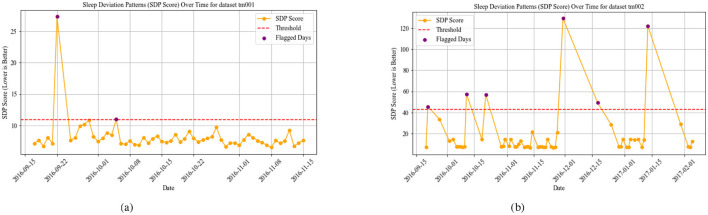
SDP using SOD, SDD, SII, and SCI in datasets TM001 and TM002. **(a)** SPD for TM001. **(b)** SPD for TM002.

**Figure 5:**
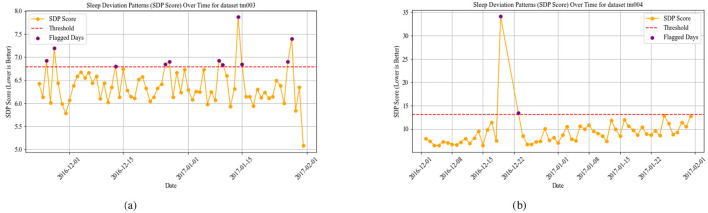
SDP using SOD, SDD, SII, and SCI in datasets TM003 and TM004. **(a)** SPD for TM003. **(b)** SPD for TM004.

**Figure 6:**
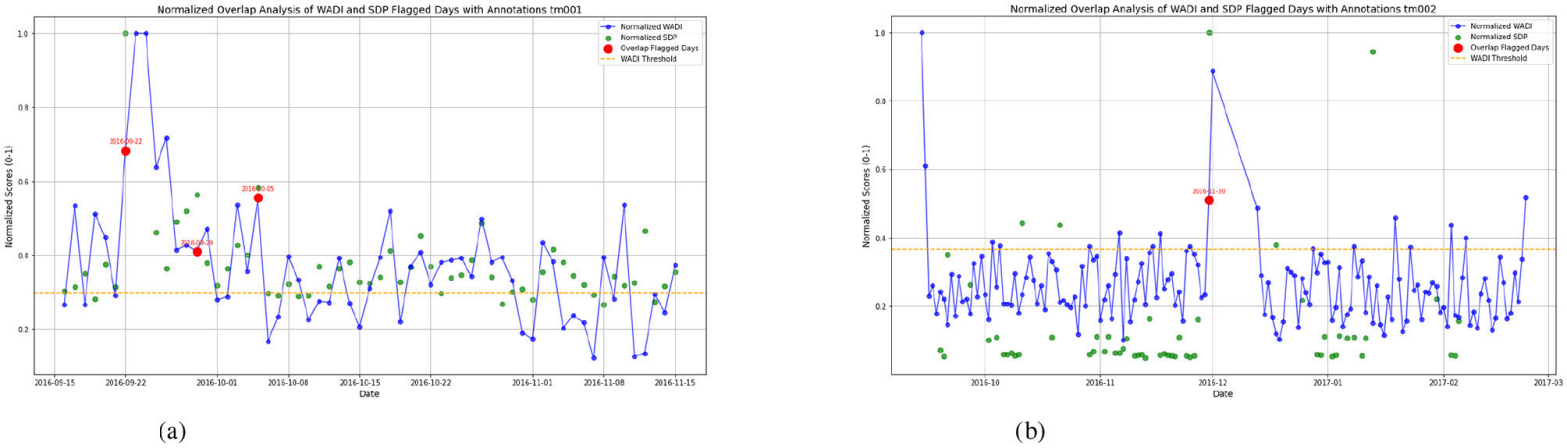
**(a)** Overlap of WADI and SDP for TM001. **(b)** Overlap of WADI and SDP for TM002.

The WADI result for datasets TM003 in Figure 3a and TM004 in Figure 3b display higher values, suggesting significant disruptions in both activity and sleep cycles. Increased WADI means that the persons activity patterns deviate substantially from expected daily rhythms, possibly reflecting disorganized behavior, restlessness, or prolonged inactivity. Our comparison shows that TM004 exhibits a higher frequency of flagged days (red points) than TM003, suggesting greater deviations from the reference routine in TM004. Specifically, TM004 shows more pronounced spikes and variability in the WADI values, while TM003 remains relatively stable with fewer threshold exceedances. This divergence suggests that tm004 may have more unusual or irregular activity patterns, which could be because of environmental factors, changes in user behavior, or problems with the sensors. Figures 4, 5 show the Sleep Deviation Patterns (SDP scores) for datasets TM001, TM002, TM003, and TM004 over time. Here, TM003 has more irregular SDP scores, with several peaks going beyond the predefined threshold, which means there are more flagged days. This shows anomalous patterns in sleep metrics, including delays in falling asleep, waking up, and sleep duration. On the other hand, TM004 has less variance, which means that sleep patterns are more stable. However, despite this consistency, the overall sleep duration in TM004 is remarkably shorter and remains below the average range observed in TM003. This indicates that while the sleep behavior in TM004 may be stable, it may still reflect suboptimal or insufficient sleep, increased night awakenings, and possibly reversed sleep-wake cycles. All these are known early warning signs of dementia, Parkinsons disease, and other neurodegenerative conditions which could be clinically relevant for early detection of neurodegenerative symptoms. The comparison emphasizes the importance of analyzing both variability and absolute values when assessing sleep quality across individuals. The overlap of WADI and SDP as depicted in Figure 6 also shows some common anomalies in the dataset TM001 and TM002. Results for dataset TM004 (Figures 3 and **8**), the individual exhibits the highest levels of activity and sleep irregularity, potentially indicating severe cognitive impairment or even undiagnosed neurodegenerative pathology.

### 5.6 WADI and SDP as indicators of neurodegenerative symptoms

#### 5.6.1 Activity deviations (WADI) and their association with cognitive health

Higher WADI values in TM003 and TM004 suggest that these individuals experience activity irregularities linked to cognitive decline. Disruptions in daily activity cycles (such as erratic movement, long inactive periods, or agitation) are common in Alzheimers disease (AD), Parkinsons disease (PD), and dementia with Lewy bodies (DLB). Possible reasons for high WADI scores: Disorganized behavior: Individuals may forget planned tasks, leading to unstructured activity patterns. Apathy or restlessness: Neurodegenerative diseases often cause reduced motivation or, conversely, increased agitation and wandering, contributing to high WADI. Circadian rhythm disruption: Early-stage dementia patients may experience changes in their internal biological clock, resulting in unusual spikes in activity at inappropriate times.

#### 5.6.2 Sleep disruptions (SDP) and link to neurodegeneration

Elevated SDP values in TM003 and TM004 indicate significant sleep disturbances. Sleep problems such as insomnia, increased night awakenings, and excessive daytime sleepiness are among the earliest symptoms of neurodegenerative diseases. Possible reasons for high SDP scores: Insomnia and fragmented sleep: Frequently observed in Alzheimers and Parkinsons disease. REM Sleep Behavior Disorder (RBD): Found in Parkinsons and dementia with Lewy bodies, where individuals may physically act out dreams. Circadian rhythm dysregulation: Neurodegeneration often affects the brains sleep-wake cycle, leading to nighttime wandering, early morning awakenings, or daytime napping.

### 5.7 Correlation between unusual activity patterns and potential health risks

**Figure 7:**
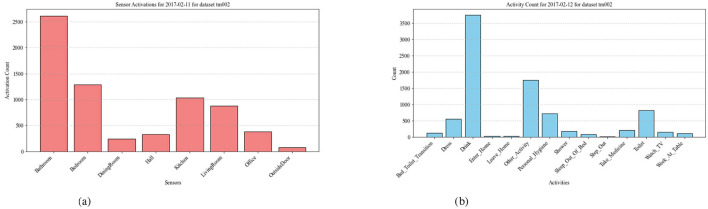
**(a)** Sensor Activation Count for TM002. **(b)** Sensor Activity Count for TM002.

**Figure 8:**
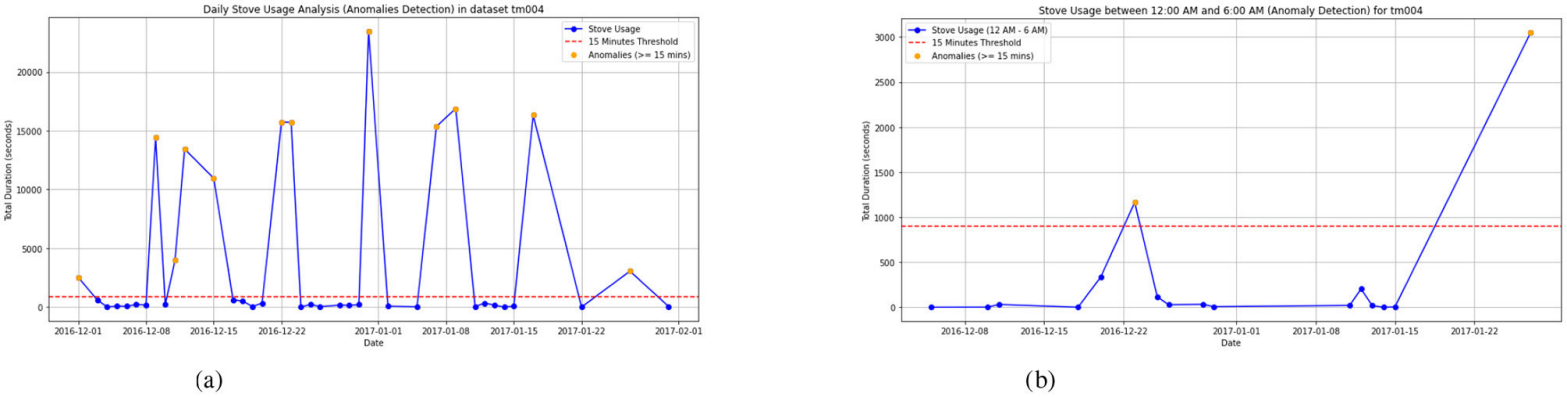
Stove usage to detect anomalies and fall detection in neurodegenerative disease. **(a)** Daily stove usage for TM004. **(b)** Night time stove usage for TM004.

Anomalies in smart home activity patterns can serve as indicators of potential health concerns. For instance, an increase in the frequency of certain activities, such as excessive bathroom visits, as depicted in Figure 7, followed by increased hydration in Figure 7, may suggest underlying health issues like urinary tract infections or dehydration. Similarly, patterns related to fall incidents in smart home datasets may serve as early indicators of underlying neurological conditions such as Parkinsons disease. While smart home systems cannot directly detect falls, unusual activity patterns–like prolonged nighttime stove usage, as seen in Figure 8 may signal disruptions in a residents routine, such as poor sleep or cognitive changes. Identifying these correlations through smart home data can provide early insights into residents' well-being, enabling timely intervention and proactive healthcare support. The analysis of sleep duration and nighttime stove usage reveals a strong correlation between sleep disturbances and unusual nighttime activity. On December 23, 2016, when stove usage exceeded 1,163 s, sleep duration was critically low at around 2 hours, indicating severe sleep disruption. Similarly, on January 27, 2017, with 3051 seconds of stove usage, sleep duration remained below 5 hours, suggesting restlessness or fragmented sleep. These anomalies, especially when observed over longer periods, may indicate underlying neurological conditions such as Parkinsons disease, where motor impairments, gait disturbances, and cognitive decline can lead to sleep issues and nighttime wandering. While falls themselves cannot be detected, such patterns can signal changes that warrant closer monitoring and could prompt caregivers to check in on the resident, potentially providing insights into their health and well-being.

### 5.8 Clinical significance

These findings carry important clinical implications. First, monitoring WADI and SDP in at-risk populations (such as older adults or those with genetic predisposition) could enhance early detection of neurodegenerative disorders. Traditional clinical evaluations might not catch gradual changes in sleep or activity, but wearable devices and smart home sensors can continuously track these indices. The data from TM001-TM004 illustrate how deviations in such metrics correlate with symptom severity. If a patients WADI and SDP begin trending upward from their baseline, physicians could be alerted to investigate further, potentially administering cognitive tests, neurological exams, or biomarker assessments earlier than they otherwise would. Early intervention is crucial in conditions like AD, where emerging therapies (e.g., anti-amyloid drugs) are thought to be most effective in the very early (pre-dementia) stage (Staff, 2025). By catching the behavioral signatures of neurodegeneration sooner, we stand a better chance of slowing disease progression. This approach is in line with the growing emphasis on digital biomarkers for dementia: subtle changes in gait, speech, sleep, and daily rhythms captured by devices may herald brain changes before traditional clinical symptoms are obvious.

### 5.9 Limitation

Our work presents a great potential for early neurodegenerative disease detection using smart home sensors, but it still faces several limitations. Sensor data often lacks clinical context, making it difficult to differentiate between normal aging patterns and early signs of neurodegeneration. Variability in individual routines and living environments can affect model generalizability. Privacy and ethical concerns may limit long-term monitoring. Moreover, slight cognitive or behavioral changes might not be captured accurately by non-invasive sensors, requiring complementary clinical assessments.

### 5.10 Future research directions

- Longitudinal analysis of individual households: future studies should examine long-term trends within each household or living lab to detect whether these deviations worsen over time, indicating progressive cognitive decline. By addressing the need for continuous data, the framework will enable a more accurate differentiation between behavioral anomalies and contextual factors, advancing the field of neurodegenerative disease monitoring and early detection.
- Integration of additional biomarkers: combining WADI and SDP with physiological markers (e.g., heart rate variability, movement tracking, EEG sleep patterns) could improve early disease detection.

## 6 Conclusion

This paper introduces a comprehensive framework for the early detection of neurodegenerative diseases by analyzing deviations in sleep and activity behaviors using smart home sensor data. By leveraging the SDP and the WADI, the proposed methodology enables passive, non-invasive, and continuous monitoring of behavioral changes that may signify early stages of cognitive or motor decline. The SDP framework captures fluctuations in sleep onset, duration, interruptions, and overall consistency, while WADI quantifies deviations in daily activities, accounting for the varying significance of different tasks. Our analysis of four real-world smart home datasets (TM001-TM004) revealed a clear behavioral stratification: TM001 and TM002 displayed stable patterns with SDP scores below 0.2 and WADI scores under 0.02, consistent with cognitively healthy routines. In contrast, TM003 and TM004 exhibited elevated SDP scores above 0.4 and WADI scores exceeding 0.03, with TM004 showing anomalies on 28% of observed days, reflecting disruptions such as fragmented sleep and irregular activity, both of which are documented early indicators of neurodegeneration.

These findings validate the potential of SDP and WADI as digital biomarkers for identifying subtle behavioral shifts long before clinical symptoms manifest. Unlike traditional diagnostic approaches that often detect the disease after significant neuronal damage, this framework allows for proactive health monitoring in a person's natural environment without reliance on wearable devices or intrusive testing. Moreover, this research supports the scalability of smart home-based solutions as part of integrated healthcare systems for aging populations. It aligns with global efforts toward preventive care, remote patient monitoring, and AI-assisted health analytics. Future work will focus on enhancing model generalizability, integrating physiological data (e.g., heart rate, sleep stages), and performing longitudinal studies to establish predictive thresholds for individual health trajectories. Ultimately, the DNA framework demonstrates significant promise in facilitating early intervention, reducing diagnostic delays, and improving outcomes for individuals at risk of neurodegenerative diseases.

## Data Availability

The original contributions presented in the study are publicly available. This data can be found here: https://casas.wsu.edu/datasets/.
